# Calumenin, A Calcium‐Binding Modulatory Protein, Effective in Pathological Calcifications and Cancers, With Therapeutic Application Promise

**DOI:** 10.1096/fba.2025-00106

**Published:** 2025-10-13

**Authors:** Parinaz Nasri Nasrabadi, Babak Jahangiri, Zahra Amiri, Forouzandeh Mahjoubi, Fatemeh Masoumi, Alireza Zomorodipour

**Affiliations:** ^1^ Department of Molecular Medicine Institute of Medical Biotechnology, National Institute of Genetic Engineering and Biotechnology Tehran Iran; ^2^ Department of Medical Genetics Institute of Medical Biotechnology, National Institute of Genetic Engineering and Biotechnology Tehran Iran

**Keywords:** γ‐carboxylation, Ca deposition, Ca^2+^‐binding, calcium ion homeostasis, CALU isoforms, calumenin, cancer, endoplasmic/sarcoplasmic reticulum‐related processes, ER stress

## Abstract

Calumenin (CALU), a multifunctional E‐F‐hand protein of the CREC family involved in various cellular processes, is ubiquitously expressed in almost all human tissues, although its expression level is tissue‐specific. CALU plays an effective role in calcium (Ca) homeostasis throughout various Ca2^+^‐related processes, and its correlations with various ER‐related functions are evident. Due to its propensity to bind Ca, participation in calcium ion (Ca^2+^)‐dependent activities, major cellular processes, such as the production and maintenance of extracellular matrix, have been attributed to this multifaceted protein. In this regard, CALU's association with various normal calcification processes, as well as pathologic Ca deposits, is apparent. Additionally, its key role as a crossroads among various normal cellular processes and many inflammatory diseases and cancers is also evident. The important correlation of CALU with cancer‐related proteins through its regulatory role in γ‐carboxylation of known cancer‐related vitamin K‐dependent proteins has also been noted. The opposing and pleiotropic functions of the CALU isoforms might play balancing roles in establishing a state of equilibrium in the cell. Given the contradictory functions of CALU isoforms during cancer, the need for a balance between these isoforms, as well as the existence of mechanisms to regulate their ratio in normal cells, is speculated. The relationship between CALU and immune response, tumor‐infiltrating immune cells, and cancer patients' responsiveness to various cancer therapies is also described. In this regard, the involvement of the CALU isoform in response to cancer treatment and various immune pathways is discussed. This comprehensive review addresses the outstanding features and the latest findings on CALU's molecular aspects and diverse functions in various physiological processes and pathological conditions.

## Graphical abstract

Calumenin (CALU) is effective in calcium homeostasis, involved in various cellular processes, ER‐related functions, normal calcification processes, pathological calcium deposits, inflammatory diseases, and cancers. Additionally, it has been linked to cancer by controlling the γ‐carboxylation of vitamin K‐dependent proteins linked to cancer. The conflicting functions of its isoforms point to its significance in maintaining cellular state of equilibrium. The association of CALU and immune response, tumor‐infiltrating immune cells, and response to various cancer therapies has also been speculated.

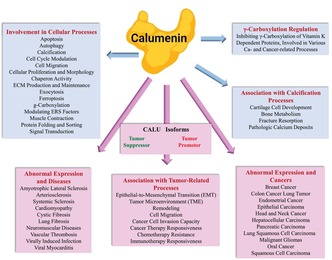

## 1 Background

### 1.1 CREC Protein Family

The CREC protein family is named after four low‐affinity calcium (Ca^2+^)‐binding proteins that possess multiple EF‐hand motifs: Cab45 (45‐kDa Ca^2+^‐binding protein), reticulocalbin (RCN), ERC‐55 (ER Ca^2+^, 55 kDa), and Calumenin (CALU) [1, 2, 3]. The EF‐hand motif is characterized by a Ca^2+^‐binding region within the protein, consisting of a helix–loop–helix structure and a consensus sequence [4]. These proteins primarily associate with Ca^2+^ and processes related to secretory pathways [2]. Members of the CREC family are retained in the lumen of the endoplasmic reticulum (ER) and the Golgi apparatus through a tetrapeptide retention signal located at their carboxyl terminus, which has been identified in both mammalian cells [5] and yeast [6], corresponding to Lys‐Asp‐Glu‐Leu and His‐Asp‐Glu‐Leu, respectively. Furthermore, the regulatory functions of CREC family members are evident in numerous critical processes, including cell proliferation, transformation, senescence, homeostasis, thrombosis, and modulation of the complement system in response to infections [3, 7]. Supporting these findings, it has been demonstrated that homozygous deletion of the *Rcn* gene is lethal in mice [8]. Additionally, dysregulation of CREC proteins in various diseases, such as cancer, neuromuscular disorders, and cardiovascular diseases, as well as in response to different forms of treatment and stress, has rendered them potential biomarkers for the diagnosis and treatment of a range of conditions [3].

In the following sections, we will briefly introduce the four known members of the CREC family, focusing on the key features of CALU and recent findings related to its molecular aspects, diverse functions in various physiological processes, and its associations with ER‐related functions and Ca‐related processes. We will also address variations in CALU expression under different cellular and physiological conditions, as well as in pathological calcifications and cancer. Additionally, we will explore CALU as a hallmark of epithelial‐mesenchymal transition (EMT) and consider the potential role of its isoforms in establishing cellular equilibrium.

#### 1.1.1 CREC Family Members

The CREC protein family includes Cab45, RCN, ERC2, and CALU, which are encoded by the *rcn1/rcn3*, *rcn2*, *sdf4*, and *calu* genes, respectively.

Cab45 is a Ca^2+^‐binding CREC protein in the Golgi lumen [3]. This protein, with three variants, namely Cab45‐G, Cab45‐C (a 130 aa truncated form of Cab45‐G), and Cab45‐S (a secreted form), is expressed ubiquitously in all tissues and is encoded by the *SDF4* gene, containing seven exons [9]. The 362 amino acids of Cab45 precursor have a signal sequence, six EF‐hands, and a C‐terminal HEEF. Cab45 is required for Ca^2+^ import into the trans‐Golgi network (TGN) by binding to the secretory cargo in a Ca^2+^‐dependent manner [10].

RCN, another member of the CREC family, is an ER‐resident Ca^2+^‐binding protein that is also expressed on the cell surface [11]. The RCN variants are encoded by a seven‐exon gene [12]. Two major types of RCN, namely, RCN1 and RCN3, are 331 and 328 amino acids, respectively, each containing a signal sequence, six EF‐hands, and an HDEL retention signal. RCN1 is involved in tumorigenesis and tumor progression and has been suggested to be required for cell proliferation and migration [13]. RCN2 is the third member of the CREC family, is encoded by the *RCN2* gene, and consists of a signal peptide, 6 EF‐hands, and an HDEL retention signal [14]. It has been shown that RCN2 is involved in various diseases and abnormal cell behavior, and its interaction with cytosolic proteins, vitamin D receptors, and many other proteins illustrates that it plays a remarkable role in immunity, redox homeostasis, regulation of the cell cycle, and coagulation [15]. Extracellular signal‐regulated kinase 2 (ERK2) regulates the EGFR‐ERK pathway, which plays a crucial role in cell proliferation and tumor growth [16]. More recently, ERC2 has also been introduced as a candidate biomarker to identify atherosclerosis patients [17].

CALU, the central subject of this review, is the fourth member of the CREC family. It is a multifunctional Ca2+‐binding protein, participating in a variety of physiological processes, particularly several ER‐related functions. CALU is conserved from worm to human, ubiquitously expressed in all human tissues, and highly expressed in cardiomyocytes in the heart, smooth muscle, lung, and placenta [1, 18, 19, 20, 21, 22, 23]. The hCALU undergoes a dramatic structural change upon Ca binding, from an unstructured to a structured state [24]. The structure of CALU reversibly changes from an unfolded state at low Ca2+ concentrations to a predominantly alpha‐helical state at higher Ca2+ concentrations [25]. This structural transition, which plays a key role in cellular Ca homeostasis, is influenced by Leu15 [2]. This feature distinguishes vertebrate CALU from its invertebrate homologs, which have a stable structured state, even in the absence of Ca [24]. The hCALU has both intracellular and extracellular functions [2, 5, 18].

## 2 Main Features of CALU

### 2.1 The Human CALU Genetics and Structure

In both humans and mice, there are two copies of the CALU gene, each containing six exons, and in humans, both genes map to chromosome 7q32 [21]. Each of the CALU genes consists of five identical exons (exons 1, 3, 4, 5, and 6) and a different exon 2. The hCALU is a 37‐kDa protein, consisting of 315 amino acids, which includes an amino‐terminal signal sequence, six EF‐hand motifs, a potential N‐glycosylation site at Asn‐131, and a C‐terminal ER retention signal consisting of the amino acid sequence His‐Asp‐Glu‐Phe (HDEF) [3]. The hCALU, due to its charged and flexible structure, low hydrophobicity, and dynamic Ca^2+^‐dependent conformational changes, exhibits typical features of intrinsically disordered proteins (IDPs) [26]. IDPs, with highly disordered sequences, generally lack bulky hydrophobic amino acids and are therefore unable to form any tertiary structure with a regular hydrophobic core [27]. CALU was also identified as a target for tyrosine phosphorylation [28].

Two CALU isoforms (i.e., CALU1 and CALU2) were first identified by Menon and Omenn (2010) [29]. Years later, it was reported that alternative splicing of hCALU pre‐mRNAs leads to the production of 15 isoforms (CALU1‐15) [30], among which isoforms 1 and 2 were the most abundant [18, 31]. The CALU isoforms 1 to 14, with N‐terminal signal peptides, are localized in the lumen of the membrane system as well as in the extracellular space, and CALU15, which lacks a signal peptide, translocates between the nucleus and cytoplasm through interacting with essential players in metastasis such as importin α, *Crm1*, *and Ras‐related nuclear protein GTPase* (Ran‐GTPase) [30]. Importin α is an adaptor protein involved in the import of proteins into the cell nucleus [32]. Crm1 is a major transport receptor for the export of proteins out of the nucleus [33], and Ran‐GTPase, a member of the Ras superfamily, cycles between an active (GTP‐bound) and inactive (GDP‐bound) state [34]. For CALU15 to enter the nucleus, its phosphorylation at Thr‐73 is necessary [30].

### 2.2 Contribution of CALU to Diverse Cellular Processes

**FIGURE 1:**
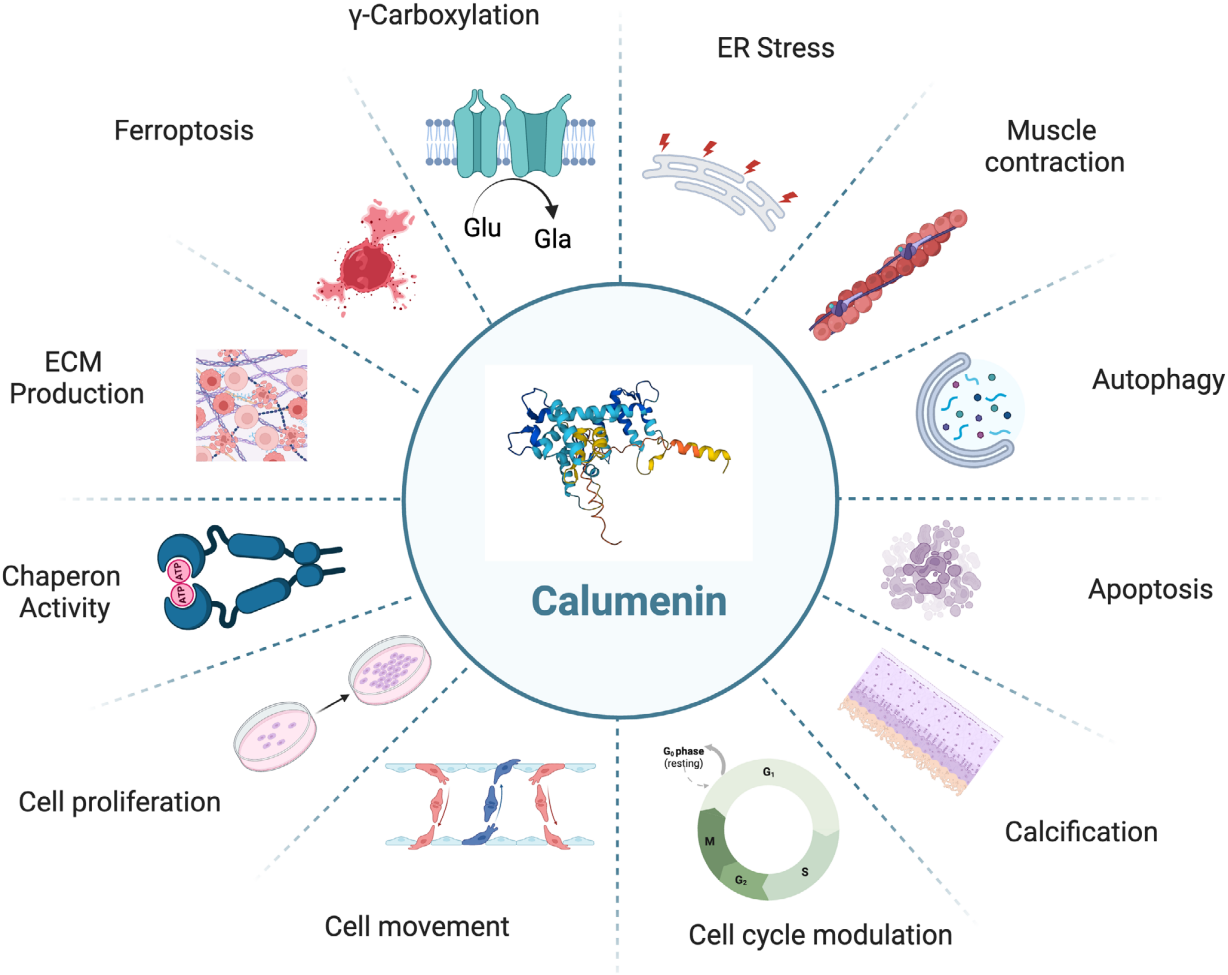
The main cellular processes in which CALU participates. In cellular physiology, CALU participates in a multitude of processes, contributes to various functions crucial for cellular homeostasis and regulation. These processes encompass ER stress response, muscle contraction, autophagy, apoptosis, calcification, cell cycle modulation, cell migration, proliferation, chaperone activity, extracellular matrix production, ferroptosis, and γ‐carboxylation. CALU's involvement in these diverse cellular activities underscores its essential roles in maintaining cellular integrity, protein quality control, and signaling regulation. Dysregulation or dysfunction of CALU can have profound implications for physiological processes and may contribute to the pathogenesis of conditions such as cancer, neurodegenerative diseases, and metabolic disorders.

The main cellular processes in which CALU is involved are depicted in Figure 1. CALU, with a low affinity, binds up to 7 Ca ions and has distinctive roles in various Ca^2+^‐related processes, such as chaperone activities in protein‐correct structure modeling and maturation [35, 36], ER‐stress (ERS) [35], signal transduction, contraction, and inhibition of γ‐carboxylation of vitamin K‐dependent proteins (VKDPs) [7, 37, 38, 39, 40, 41]. It is also involved in bone metabolism, chondrocyte growth, and various calcification processes with normal or pathological Ca deposits, all of which affect cellular Ca^2+^ concentration [42]. Therefore, CALU has been known as a major player in cellular Ca recycling and homeostasis [43, 44, 45].

CALU participates in a variety of ER‐related processes, such as exocytosis, autophagy [46, 47], apoptosis [35], ferroptosis [48], cell cycle modulation [49], and cell proliferation and morphology [2, 3, 36]. CALU also modulates cellular stress by regulating GRP78, phosphorylated PERK, C/EBP homologous protein (CHOP), p‐JNK, and antiapoptotic Bcl‐2 [35].

The implication of CALU in extracellular matrix (ECM) production and maintenance, and its participation in cancer‐related processes, such as tumor microenvironment (TME) remodeling, chemotherapy resistance, and cell migration [30, 50, 51, 52], was also shown. The CALU‐reported roles in cell cycle, migration, and the cancer cell invasion capacity are attributed to cytoskeleton remodeling, filopodia formation, extracellular signal‐regulated protein kinases 1 and 2 (ERK1/2) pathway regulation, and the protection of ECM protein fibulin‐1 against metalloprotease degradation [7, 30, 40].

Association with immune pathways is another outstanding feature of CALU. In this regard, its relationships with the infiltration of immune cells into heart tissue [53], tumor‐infiltrating immune cells, multiple immune checkpoint‐related genes (ICRGs) [48, 54, 55], and cancer therapy responsiveness [56] are worth mentioning. In the following sections, the main CALU activities will be discussed in more detail.

#### 2.2.1 Interactions Between CALU and Calcium

Calcium ion is among the most important and common second messengers inside the cell, whose concentration changes affect cellular processes such as fertilization, proliferation, differentiation, muscle contraction, nerve signaling, secretion, exocytosis, chemotaxis, synaptic transmission, ciliary movement, enzyme activation, growth, and development [2, 57, 58]. Although the presence of Ca^2+^ is necessary for these processes, it should be kept in mind that excess Ca^2+^ can also be very harmful to the cell [59]. It is known that Ca^2+^ homeostasis is generated and maintained through coordinated functional molecules associated with the plasma membranes of cellular compartments, such as the ER, where the storage and release of Ca^2+^ have impacts on critical physiological functions [60, 61]. Dysregulation of Ca^2+^ homeostasis in the ER activates the unfolded protein response, a pathway that attempts to restore cellular homeostasis upon exposure to ERS, ultimately leading to apoptosis and cell death [62, 63].

**FIGURE 2:**
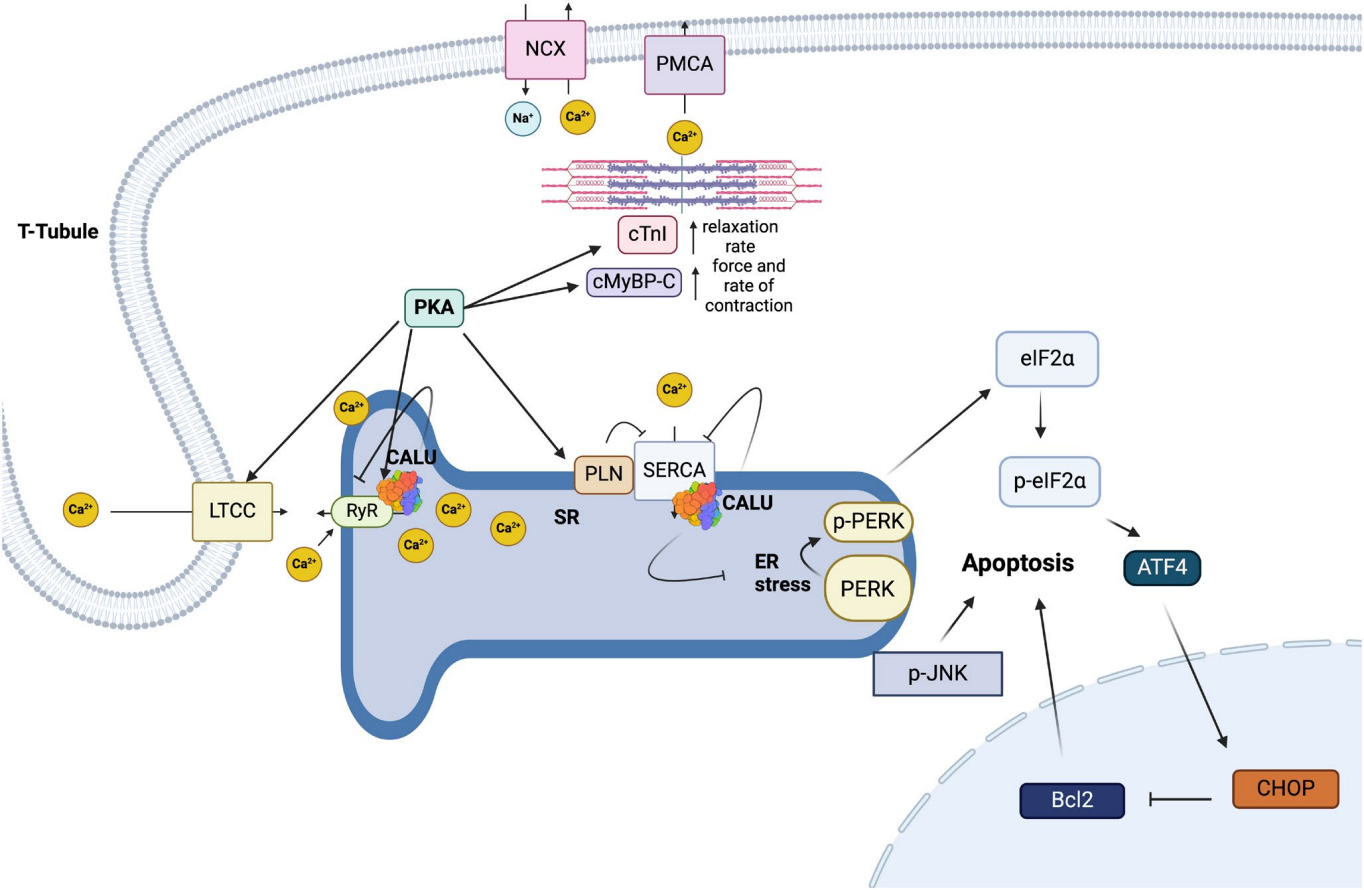
A hypothetical model for the role of CALU, located in the SR lumen, in the ER stress response, the PERK/Bcl2 apoptotic pathway, and the regulation of excitation–contraction coupling in the heart. The SR is pivotal in the Ca^2+^ cycling of muscle cells, where Ca^2+^ release from the SR by RYR initiates muscle contraction, and Ca^2+^ uptake into the SR by the SER Ca^2+^‐ATPase (SERCA) leads to muscle relaxation. Recent evidence suggests that CALU directly binds both RYR and SERCA within the SR lumen. CALU overexpression diminishes the rates of Ca^2+^ release and uptake due to its inhibitory effect on RYR and SERCA, respectively, whereas CALU knockdown enhances these rates by activating RYR and SERCA. CALU regulates Ca^2+^ release from the SR, interacting with signaling regulator proteins, notably SERCA2a and RYR, thereby modulating their functions. The interaction of CALU with SERCA2 in the cardiac SR supports its role in regulating Ca^2+^ uptake during excitation–contraction, both in smooth muscle and cardiac muscle tissues. This regulatory mechanism illustrates CALU's importance in fine‐tuning the intricate balance of Ca^2+^ handling critical for proper cardiac function.

Some EF‐hand proteins, with high affinities for Ca^2+^, including Troponin C, Calmodulin, Parvalbumin, and intestinal Ca^2+^‐binding proteins, are generally involved in Ca^2+^ regulation in the cytosol [64]. Similarly, CALU, with a low affinity for Ca^2+^, acts as a key regulator in ER‐Ca^2+^ homeostasis [18, 36, 65]. CALU regulates Ca^2+^ release from the sarcoplasmic reticulum (SR), where it interacts with signaling regulator proteins, in particular the SR Ca^2+^‐ATPase (SERCA2a), a Ca^2+^ pump implicated in SR‐Ca^2+^ refilling [43, 44, 45], and the ryanodine receptor (RYR), which releases Ca^2+^ from the SR to induce muscle contraction [44, 66], thereby regulating its functions [53] (Figure 2).

CALU involvement in Ca^2+^ regulation in the ER was demonstrated by siRNA‐mediated inhibition of CALU, which led to an increased concentration of free Ca^2+^ in ER, while CALU overexpression decreased the free Ca^2+^ concentration in the ER [36]. Therefore, CALU has been introduced as a major player in cellular Ca recycling and homeostasis [2, 44].

CALU has a crucial function in ERS in programmed cell death, as evidenced by its substantial impact on cellular Ca^2+^ homeostasis on the one hand and the occurrence of apoptosis in the ER after changes in Ca^2+^ homeostasis on the other (Figure 2). It has previously been proposed that CALU modifies cellular stress via controlling proapoptotic proteins, ERS factors, and antiapoptotic Bcl‐2 [35]. While the regulatory function of CALU on Ca^2+^ homeostasis was established, the impact of changes in Ca^2+^ ion concentration on the conformation and function of CALU was also evident. The CALU conformation reversibly changes from unfolded at low Ca^2+^ concentrations to a compact‐helical state at high Ca^2+^, thereby acting as a Ca^2+^ sensor [25]. Since CALU is a Ca^2+^‐binding protein, similar to other chaperones in ER/SR, its chaperone activity was suggested to be regulated by Ca^2+^ concentration (see Section 2.2.2) [33]. It is worth mentioning that there are other Ca^2+^‐binding proteins in the ER with low affinity and high capacity, such as calsequestrin, BiP, endoplasmin, Erp72, PDI, and calnexin, which are usually activated following an increase in Ca^2+^ concentration [67]. Therefore, close cooperation between such proteins and Ca^2+^ concentration is required.

##### 2.2.1.1 CALU and Calcification‐Related Processes

Among the processes in which CALU affects Ca^2+^ concentration are the calcification‐related processes, such as bone metabolism and vascular calcification [42], thrombosis, and atherosclerosis [41, 68, 69], chondrocyte development [70], fracture resorption [71], lung fibrosis [72], and many other pathological calcifications outlined in Section 3.1.

##### 2.2.1.2 CALU Regulates γ‐Carboxylation of VKDPs

CALU interacts with the components of the γ‐carboxylation system by targeting both VKOR and γ‐carboxylase (γC), inhibiting the γ‐carboxylation of the VKDPs' glutamate and inhibiting their γ‐carboxylation [7, 37, 38, 39, 41]. By regulating the γ‐carboxylation of various VKDPs, functionally, CALU interacts indirectly with proteins involved in Ca^2+^ homeostasis and signaling regulation, in particular SERCA2a, a calcium pump implicated in SR‐Ca^2+^ refilling [4, 44, 73], and the RYR [66], thereby regulating their functions. Taking a small interfering (siRNA)‐mediated knockdown approach, the inhibitory effect of CALU on the γ‐carboxylation of FIX was demonstrated for the first time [74]. We also demonstrated the improvement of rhFIX's functional expression after CALU downregulation, intending to improve rhFIX's γ‐carboxylation in a mammalian host cell expression system [75].

**FIGURE 3:**
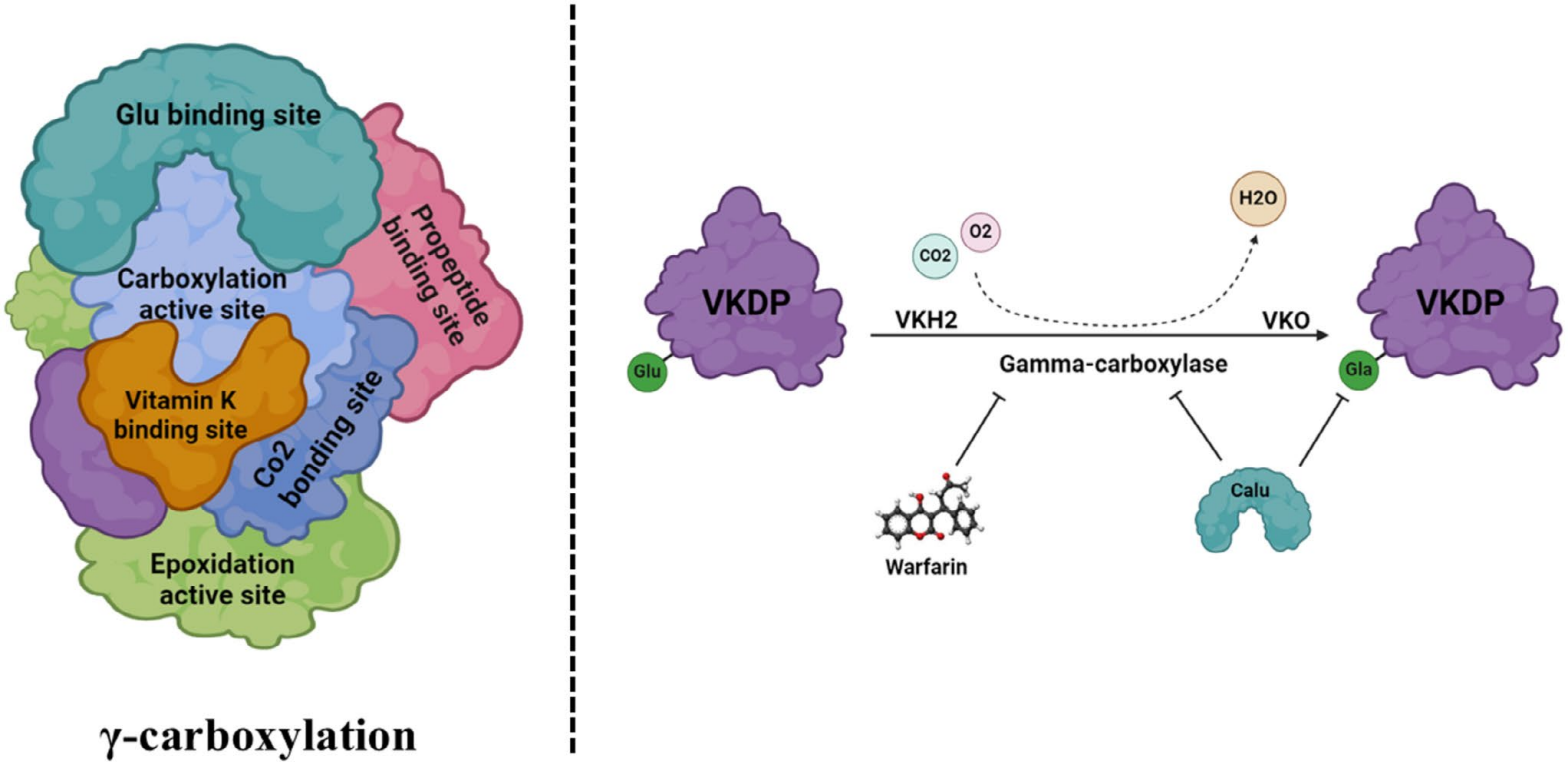
Carboxylation of VKDPs is a necessity for their functionalization. The vitamin k dependent γ‐carboxylation of specific Gla residues in the N‐terminal region of a VKDP is essential for membrane binding activity. Additional γ‐carboxyl groups on Gla residues result in highly negatively charged side chains that allow calcium to bind to VKDP. Carboxylation requires the abstraction of a proton from the 4‐carbon of glutamate by reducing vitamin K, resulting in the conversion of vitamin K to vitamin K epoxide, which in turn must be recycled back to vitamin K before being reused. This reaction is catalyzed by the enzyme VKOR. CALU interacts with the components of the γ‐carboxylation system by targeting both VKOR and γC, inhibiting the γ‐carboxylation of the glutamate residues of VKDPs and inhibiting their γ‐carboxylation.

The impact of CALU on the Ca^2+^ concentration can occur via its regulatory role on the γ‐carboxylation of VKDPs [3, 76, 77, 78, 79]. The vitamin K‐dependent (VKD) γ‐carboxylation of particular glutamic acid (Gla) residues in a VKDP's N‐terminal region is essential for CALU's biological activity [78]. The extra γ‐carboxyl groups in the Gla residues result in highly negatively charged side chains, allowing Ca to bind the VKDP [77]. An 18‐amino acid propeptide, N‐terminal to the Gla domain, acts as a recognition site for γC [77, 80]. A major feature of a VKDP is its membrane‐binding activity, which is generated as a result of a highly negatively charged carboxyl side chain and its tendency for Ca‐binding, allowing the protein to interact with the negatively charged phospholipid membranes [78, 80, 81]. Carboxylation requires the abstraction of a proton from glutamate by reduced vitamin K and ends with the conversion of vitamin K to vitamin K epoxide (Figure 3). The vitamin K epoxide must be recycled to vitamin K before it can be reused, a reaction catalyzed by vitamin K epoxide reductase (VKOR) [82].

To date, 17 VKDPs have been identified and implicated in a broad range of functions [83, 84]. VKDPs typically require nine to thirteen Glu residues at the Gla domain to be properly carboxylated [85]. When VKDPs were first identified, only the hepatic proteins of this type that are involved in coagulation, namely factors II, VII, IX, and X, and proteins C, S, and Z, were of interest to researchers [42]. However, the expansion of knowledge led to the identification of other VKDPs that are involved in extrahepatic activities, often associated with various processes such as vascular calcification, bone metabolism, inflammation, carcinogenesis, apoptosis, Ca homeostasis, mineralization, bone growth control, cell migration, angiogenesis, and signal transduction [83, 84, 86, 87, 88, 89]. Evaluation of publicly available data sets showed that γC, VKORC1, and VKORC1L1, the main enzymes mediating carboxylation, were overexpressed in 24% of BCs [82, 90]. Involvement of cancer‐related VKDPs and the impact of their γ‐carboxylation in cancers and cancer‐related processes is discussed in more detail in Section 3.2.4.

#### 2.2.2 The CALU Chaperone Activities

Molecular chaperones are proteins that mediate the correct assembly and folding of other proteins, without being components of the final functional structures [91]. The participation of CREC proteins in various chaperone activities has been shown [3]. Interaction of the RNC3 with proPACE4 [12], and interaction of RCN2 with a neurotoxin receptor [14, 92] are examples of chaperone‐like activities of the proteins of the CREC family in the secretory pathway. CALU has a low affinity for Ca and acts as a molecular chaperone that binds various proteins within the ER and is involved in modeling correct protein structure and maturation [26, 44]. The IDP‐like nature of CALU is essential for adaptive and transient interactions and cell signaling, and helps it to function as a chaperone and molecular controller of the ER. CALU is significantly more charged and less folded compared to classical chaperones, and shows lower hydrophobicity and aggregation propensity [26].

Interactions of CALU with components of secretory proteins, such as sec63p, a translocase complex [93], and fibulin‐C1, the C1 esterase inhibitor [7, 40], are examples of CALU chaperone activities. The function of CALU in reducing ERS and inhibiting ER‐initiated cellular apoptosis in neonatal rat ventricular cardiomyocytes, through the modulation of ERS proteins, has also been described as the CALU chaperone activity, which might be Ca^2+^‐dependent [35]. Another example of CALU's chaperone activity appears in the regulation and maturation of cystic fibrosis transmembrane conductance regulator (CFTR), a protein in which some mutations can cause severe cystic fibrosis (CF) [36, 73]. Read more on the CALU association with CFTR in Section 3.1.

## 3 Reported Diseases Associated With CALU Abnormal Expression

The involvement of CALU in various physiological processes, particularly its role in modulating several important Ca‐dependent activities and maintaining cellular Ca homeostasis, is evident [3]. Besides, specific variations in its expression occur in various pathophysiological conditions and diseases related to calcium deposition, such as neuromuscular and cardiovascular diseases, cancers, and following different forms of treatment and stress (Table 1). Accordingly, it has become a promising biomarker for diagnosing and treating these types of diseases. The next two sections discuss the association of CALU with two major groups of pathophysiological disorders, namely pathological calcifications and cancer, in more detail.

**TABLE 1:** Reported diseases associated with CALU abnormal expression.

Disease type	Disease names	References
**Disease, with pathological calcium deposits**	Amyotrophic lateral sclerosis (ALS)	[94]
	Atherosclerosis	[68, 69]
	Cardiomyopathy	[95]
	Cystic fibrosis	[73, 93, 95]
	Lung fibrosis	[3, 70]
	Neuromuscular diseases	[3]
	Systemic Sclerosis (an autoimmune disease)	[54]
	Vascular thrombosis	[41]
	Viral myocarditis	[96]
**Cancers**	Breast cancer	[97, 98]
	Clear cell renal cell carcinoma	[99]
	Colon cancer	[100, 101]
	Endometrial cancer	[102]
	Epithelial carcinoma	[103]
	Head and neck	[104]
	Hepatocellular and pancreatic carcinoma	[51, 105]
	Lung tumor	[100, 106]
	Lung squamous cell carcinoma	[104]
	Malignant gliomas	[107]
	Oral cancer	[108]

### 3.1 Association of CALU With Pathological Calcifications

Due to CALU's tendency to bind Ca^2+^, it is associated with pathological Ca deposits in lung fibrosis, vascular thrombosis, arteriosclerosis, and neuromuscular diseases [3, 41, 68, 69]. In this regard, CALU was also reported among the 10 genes that are differentially expressed in systemic sclerosis, which is an autoimmune disease, with pathological Ca deposits [54]. We can also refer to reports about restrictive cardiomyopathy caused by severe myocardial calcification, in which abnormal expression of CALU was reported [95].

CALU is downregulated in the cells with the mutant CFTR, compared with those with normal CFTR, which could reduce CFTR translocation [109, 110]. The inhibition of CALU results in increased CFTR activity at the plasma membrane without altering ER Ca content [36]. Tripathi et al. [26] described CALU as a charged protein exhibiting close similarity with IDPs and hypothesized CALU as a regulator of the folding of CFTR mutants. This example of the CALU chaperone activity occurs independently of ER Ca content, demonstrating the association of CALU with CF disease [73].

The interaction of CALU with SERCA2 in the rat cardiac SR supports its involvement in regulating Ca^2+^ uptake during the excitation and contraction process, both in smooth muscle, and plays an important role in maintaining normal heart function [43, 44, 45, 53] (Figure 2). There are established correlations between vascular calcification, ERS, and myocardial ischemia/reperfusion (I/R) injury [111]. ERS plays an important role in cardiac ischemia/reperfusion injury [112], and CALU was shown to relieve cardiac injury by inhibiting ERS‐initiated apoptosis during viral myocarditis [96] and cell‐induced apoptosis [113].

Amyotrophic lateral sclerosis is a fatal type of motor neuron disease, characterized by progressive degeneration of nerve cells in the spinal cord and brain, and CALU was found to be one of the proteins that is significantly dysregulated in this disease [94].

The indirect effect of CALU on Ca^2+^ concentration, through inhibiting γ − carboxylation, may lead to pathological outcomes by disrupting the functions of VKDPs like GRP and MGP, which are involved in abnormal calcium deposits [114], as described in Section 3.2.4.

### 3.2 The CALU Involvement in Cancer‐Related Processes

The CALU expression variation occurs in various cancers, including endometrial cancer [102, 106], colorectal cancer (CRC) and lung cancer (LC) [100], head and neck cancer (HNC) [104], hepatocellular carcinoma (HCC) [105], lung squamous cell carcinoma (LUSC) [115], breast cancer (BC) [97], and many others, to varying degrees, although their variations appeared to be cancer‐specific. So far, the association of CALU expression with a wide range of malignancies has been shown [3, 52, 97, 98, 116, 117]. In most of these studies, CALU was identified as a metastasis‐related protein. In several cancers, such as oral cancer [108], colon and lung cancers [101, 106], breast cancer (BC) [97, 98], more malignant gliomas [107], and in more recent works in clear cell renal cell carcinoma (ccRCC) [99] increased CALU expression was shown. In most of these studies, knockdown or aberrant expression of CALU could affect the proliferative and invasive abilities of the tumor cells, effectively reversing EMT progression and inhibiting their migration.

We also showed about 3.4 times higher CALU transcription levels in BC patients in tumor tissues than in normal adjacent tissues [118]. A higher level of CALU in metastatic human lung cancer cells than in non‐metastatic samples was shown [106]. In our previous study, we introduced CALU as a member of a panel together with the *AURKA* and *MCM2* genes, with high discriminative accuracy in dissociating the biopsies of colon and lung cancers from healthy samples, where the high expression levels of these genes are negatively correlated with the patient's survival [100]. In combination with CDH11 stromal expressions, CALU showed a significant relationship with disease‐free survival and poor prognosis, and therefore, these two proteins were proposed as stromal biomarkers with prognostic significance in colon cancer [119]. The upregulation of CALU in an irradiated epithelial carcinoma cell and its promoting role in tumor cell survival during hypoxia were also demonstrated [103]. A strong correlation of CALU with hypoxia was demonstrated through cluster analyses in glioma [107]. In contrast, downregulation of CALU in metastatic cell lines of the HNC [104, 120], endometrial cancer [102], hepatocellular and pancreatic carcinomas [52, 105], and lung squamous cell carcinoma [115] was evident. So far, no adequate explanation for the cancer‐specificity of CALU variations has been provided. However, this can be attributed to the tissue specificity of CALU expression as well as the expression diversity of its isoforms in different cellular conditions, under the influence of the tumor microenvironment (TME). A recent study confirmed that CALU promotes lung adenocarcinoma progression by increasing cell proliferation, migration, and invasion [121]. Upregulation of CALU and the known cancer‐associated fibroblast (CAF) markers, such as Periostin (POSTN, or PSTN), α‐smooth muscle actin, and podoplanin, under the microRNA‐21 (miR‐21) induction, in lung cancer, suggested that the CAF‐derived CALU might function as an effector molecule in lung adenocarcinoma [122]. A dual regulatory role of CALU concerning stromal and immune components of the TME in bladder cancer and its involvement in stroma‐related pathways, including ECM remodeling, hypoxia, and angiogenesis, was also reported [48].

Collectively, the findings on CALU reported so far support its crucial role in cancer‐related processes, including the development of malignant phenotypes and cancer cell survival [107, 119], filopodia formation and cell migration [30], invasion [106], and metastasis [123]. Furthermore, CALU has major effects on cancer cell response to cancer therapies [48, 56] and resistance to chemotherapy [124], in particular.

#### 3.2.1 CALU Is Synergistic With EMT Key Markers

In 2015, taking a knockout approach, CALU was identified among metastasis‐associated proteins that facilitate lung cancer cell invasion [106]. Our group showed direct associations between CALU upregulation and tumor invasiveness in lung and colon cancers [100]. In a more recent study, in triple‐negative BC (TNBC) and other cancers, CALU was found among the top 30 upregulated genes in the CD44‐high/CD24‐low cluster, which might function as cancer stem cell regulators [125]. Based on most of the evidence mentioned above, CALU has been introduced as a hallmark of the EMT.

EMT is a primary step in the metastasis cascade, during which cells lose their epithelial characteristics and gain mesenchymal behavior [126]. Based on the data obtained from the analysis of the Cancer Genome Atlas (TCGA) and Gene Expression Omnibus (GEO) databases, and multiplex immunohistochemistry experiments, CALU was introduced as a representative EMT‐related signature and the most abundantly expressed protein in CAF in gastric cancer (GC) [127]. In a study by Yang et al. [107] a gene ontology analysis revealed a strong correlation between CALU and genes that were mainly enriched in cell/biological adhesion, response to wounding, and extracellular matrix/structural organization, all of which were strongly correlated with EMT phenotypes in glioma. In the same study, CALU was correlated with more malignant phenotypes and suggested to function synergistically with EMT markers, such as N‐cadherin, Vimentin, SNAIL, SLUG, and TWIST1. Analysis of the data from the TCGA, GTEx, and GEO databases revealed a consistent upregulation of CALU across several tumor types, including breast invasive carcinoma (BRCA), kidney renal papillary cell carcinoma (KIRP), liver hepatocellular carcinoma (LIHC), head and neck squamous cell carcinoma (HNSC), and brain lower‐grade glioma (LGG), with elevated CALU expression being associated with unfavorable prognoses [128].

#### 3.2.2 CALU‐Related EMT Signaling Pathways

Transmembrane receptors, called integrins, mediate interactions between cells and the ECM and are crucial for cell–ECM interactions and migration [129]. It has been previously hypothesized that CALU affects integrins in EMT and processes linked to TME modulation [130]. Given that CALU is involved in EMT and TME modulation, it is plausible that CALU knockdown could affect integrin expression or activation, thereby reducing cell migration. It was shown that CALU, together with fibulin‐1, also interacts with fibronectin and is dependent on both syndecan‐4 and α5β1‐integrin to suppress the ERK1/2 signaling and inhibit cell migration [52]. This cooperation also prevents fibulin‐1 degradation by MMP‐13 [51, 52].

Still affecting the ERK signaling pathway, but with an antagonistic role, CALU has also been shown to block phosphorylated ERK signaling in mucosal melanoma cells, where miRNA‐mediated CALU degradation, both in vitro and in vivo, leads to the promotion of apoptosis and cell cycle arrest while suppressing cell growth, migration, and proliferation [124].

Additionally, CALU may affect the MAPK pathway through the growth differentiation factor 15 (GDF‐15) [131, 132], which activates AKT, affecting downstream targets related to migration, proliferation, and survival of cells [133]. It is possible that CALU influences the MAPK pathway through GDF‐15 and that CALU knockdown could disrupt this signaling, leading to reduced migration [51]. The MAPK pathway is a key signaling cascade involved in cell proliferation, differentiation, migration, and apoptosis. GDF‐15 can bind to ErbB2, activate the PI3K/AKT and MAPK/ERK pathways, upregulate Cyclin D1 and Cyclin E1, and reduce p21, leading to the proliferation of cervical cancer cells [134]. The CALU knockdown's impact on cell migration may be mediated through disruptions in key signaling pathways such as integrin, MAPK, and PI3K/AKT.

Considering all the studies mentioned above and those conducted through cluster analysis, three EMT signaling pathways—TGF‐β, PI3K/AKT, and hypoxia—are strongly correlated with CALU [107]. However, further research is needed to determine the exact mechanisms of CALU's correlation with these and other signaling pathways to draw a framework for the role of CALU in regulating cell migration.

#### 3.2.3 Distinct Functions of CALU Isoforms in Cancer‐Related Processes

CALU exists in 15 isoforms (CALU 1–15), generated through alternative splicing, which exhibit opposing roles in cancer by modulating proliferation, migration, and metastasis within the same tumor types [30]. Extracellular isoforms, such as CALU1 and CALU2, stabilize the ECM to inhibit tumor progression, while nuclear CALU15 promotes tumor growth by upregulating GDF‐15. This subsection details the structural diversity of CALU isoforms, their specific roles in cancers like breast and lung, the regulatory mechanisms governing their expression, and their therapeutic potential (Table 2).

**TABLE 2:** Contrasting roles of CALU isoforms in cancer proliferation.

Isoform	Localization	Role in cancer proliferation	Key mechanisms	Cancer type examples	References
CALU 1–14	ER lumen, extracellular space	Anti‐proliferative; inhibits migration	Stabilizes fibulin‐1, suppresses ERK1/2	Breast, lung	[51, 52, 135]
CALU15	Nucleus, cytoplasm	Pro‐proliferative; promotes metastasis	Upregulates GDF‐15 (pro‐tumorigenic via TGF‐β/PI3K/AKT; context‐dependent anti‐tumorigenic via apoptosis)	Breast, lung, colorectal	[30, 136, 137, 138, 139, 140, 141, 142, 143, 144, 145]
All isoforms	Varies by isoform	Modulates tumor heterogeneity	Regulated by tra2a; CALU splicing contributes to TNBC invasiveness alongside other genes (MELK, RSRC2, etc.)	TNBC	[146, 147, 148]

##### 3.2.3.1 Structural Diversity of CALU Isoforms

The human CALU gene produces 15 isoforms, with CALU1 and CALU2 (isoforms 1 and 2) being the most abundant [18, 30, 31]. Isoforms 1–14 contain N‐terminal signal peptides, localizing them to the ER lumen or extracellular space, where they regulate ECM stability and cell migration [30]. CALU15, lacking the signal peptide, translocates between the nucleus and cytoplasm, requiring phosphorylation at Thr73 for nuclear entry via importin α, CRM1, and Ran GTPase [30, 32, 33, 34]. These structural differences drive their opposing roles in cancer, with extracellular isoforms inhibiting proliferation and CALU15 promoting it.

##### 3.2.3.2 Tumor‐Promoting Role of CALU15

**FIGURE 4:**
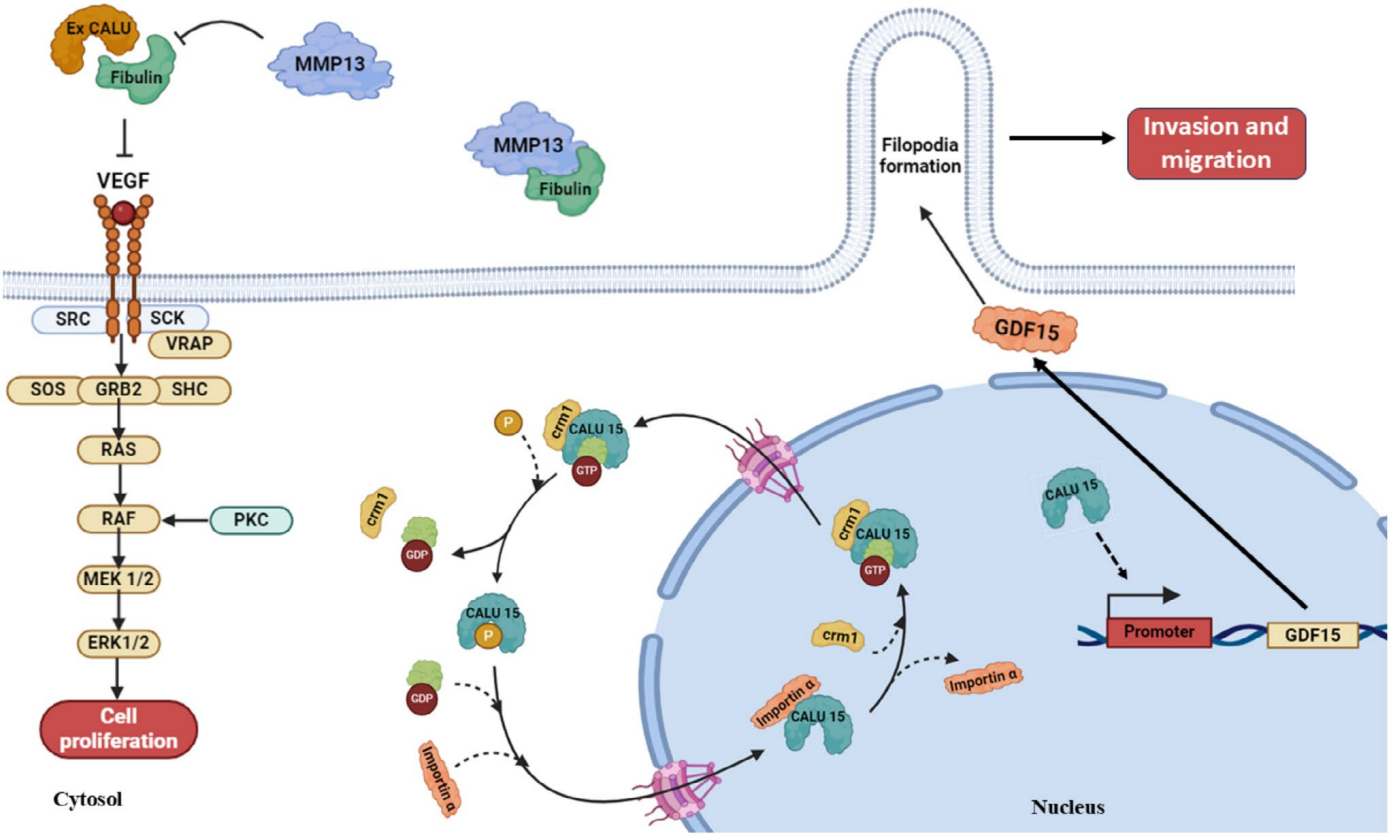
The regulatory functions of the extracellular vs. nuclear CALU isoforms in cellular processes. Extracellularly, CALU forms a complex with fibulin‐1, which plays a crucial role in inhibiting MMP‐13‐mediated degradation of fibulin‐1, thereby maintaining extracellular matrix integrity. This CALU/fibulin‐1 complex further binds to fibronectin, exerting inhibitory effects on ERK‐1/2 signaling pathways and consequent cell migration. Importantly, these inhibitory actions occur in an integrin‐ and syndecan‐dependent manner, highlighting the complex interplay between cellular adhesion molecules and signaling pathways. Additionally, CALU exhibits intracellular activity by binding to and inhibiting SERCA2 in the endoplasmic reticulum (ER), thereby modulating calcium homeostasis. This dual role of CALU, both extracellularly and intracellularly, underscores its significance in regulating cellular processes critical for maintaining tissue structure and function. CALU15, as the only nuclear isoform of CALU, promotes the proliferation of cells during tumorigenesis by upregulating GDF‐15, a cytokine of the TGF‐β superfamily, which promotes cell motility and tumor metastasis by encouraging filopodia formation.

GDF‐15, a cytokine of the GDF‐15 superfamily, is upregulated by CALU15, a phosphorylation‐dependent nuclear‐localizing CALU isoform that promotes cell motility and tumor metastasis by encouraging filopodia formation [30, 136, 137, 138, 139, 140, 141, 142, 143, 144, 145]. In several malignancies, levels of circulating serum GDF‐15 can be nearly 200‐fold higher and are associated with poor survival, suggesting GDF‐15 is a marker for cancer progression [30, 136, 137, 138, 139, 140, 141, 142, 143, 144, 145]. GDF‐15 has been shown to have both pro‐ and anti‐tumorigenic properties [30, 136, 137, 138, 139, 140, 141, 142, 143, 144, 145]. Due to its pleiotropic effects, GDF‐15 engages in a variety of cellular activities and operates under the strict regulation of many regulatory pathways (Figure 4) [30, 136, 137, 138, 139, 140, 141, 142, 143, 144, 145]. The CALU15‐promoted GDF‐15 activates TGF‐β and PI3K/AKT pathways, driving cell motility, invasion, and metastasis via filopodia formation, correlating with poor prognosis [30, 139, 149, 150]. However, GDF‐15 can also induce apoptosis in early‐stage tumors, depending on the TME [151]. Upregulation of GDF‐15 by CALU15 predominantly promotes pro‐tumorigenic effects in advanced cancers, making it a potential therapeutic target (Figure 4) (see Section 3.2.3.5).

##### 3.2.3.3 Tumor‐Suppressive Role of Extracellular Isoforms

Extracellular isoforms, particularly CALU1 and CALU2, inhibit tumor progression by stabilizing the ECM and suppressing migration [51, 52]. CALU1 forms a complex with fibulin‐1, preventing its degradation by MMP‐13 and inhibiting ERK1/2 signaling in an integrin‐ and syndecan‐dependent manner, reducing invasiveness [51, 52, 135]. CALU2, expressed in both cancerous and healthy breast tissues, similarly stabilizes ECM, counteracting EMT [135]. This opposition within a single cancer type underscores the context‐dependent roles of the isoforms.

##### 3.2.3.4 Regulation of CALU Isoform Expression

The balance of CALU isoforms is regulated by alternative splicing, mediated by the *TRA2A* gene encoding TRA2A [146, 147, 148]. In TNBC, TRA2A regulates the splicing of CALU exon 2, altering the ratio of CALU15 to extracellular isoforms, which enhances invasiveness [148]. While TRA2A also regulates other genes (e.g., MELK, RSRC2, CEACAM1, LMCD1, PALM, RFWD2), CALU's role in TNBC is supported by its correlation with GDF‐15‐driven metastatic phenotypes [30, 148]. The TME influences this balance, with upregulation of CALU15 in metastatic breast and lung cancers and extracellular isoforms in less aggressive tumors [52, 97, 98, 106, 116]. This context‐dependent regulation suggests that CALU isoform ratios play a significant role in tumor progression. However, further studies are needed to quantify the impact of TRA2A on other CALU isoform targets.

##### 3.2.3.5 Therapeutic Implications of CALU Isoforms

The opposing roles of CALU isoforms within the same cancer types offer therapeutic opportunities. Inhibiting CALU15 could suppress GDF‐15‐driven metastasis, potentially via molecules targeting its nuclear translocation [30]. Enhancing CALU1 and CALU2 could stabilize the ECM, reducing invasiveness [51, 52]. Modulating TRA2A‐mediated splicing may shift isoform ratios toward tumor suppression [148]. Additionally, CALU's regulation of VKDPs suggests isoform‐specific interventions could target oncogenic VKDPs like GAS6, enhancing therapy efficacy [152]. Further research into isoform‐specific mechanisms is essential for precision oncology.

#### 3.2.4 CALU Regulation of Cancer‐Related VKDPs' Carboxylation, A Key Topic for Future Studies

Having in mind that cancer‐related VKDPs require carboxylation for their function, and that CALU has an inhibitory role on carboxylation, it is conceivable that one way that CALU may affect the course and progression of cancer is via VKDPs' carboxylation modulation. There is a lack of direct evidence demonstrating the associations between enzymes involved in the carboxylation of VKDPs and cancer. However, in rare studies, such as the evaluation of publicly available data sets, it was shown that γC, VKORC1, and VKORC1L1, the main enzymes mediating carboxylation, are overexpressed in 24% of BCs [82, 90]. While direct experimental evidence specifically linking CALU's inhibition of VKDP carboxylation to cancer development and progression is limited, some independent studies have shown that CALU influences cancer biology and prognosis through mechanisms that likely include its regulation of VKDP activity, as well as other pathways such as tumor microenvironment remodeling and immune modulation [39, 51, 84]. Given this limited information, it appears that further research is needed to elucidate the precise pathways by which CALU affects cancer through inhibition of VKDP carboxylation.

**FIGURE 5:**
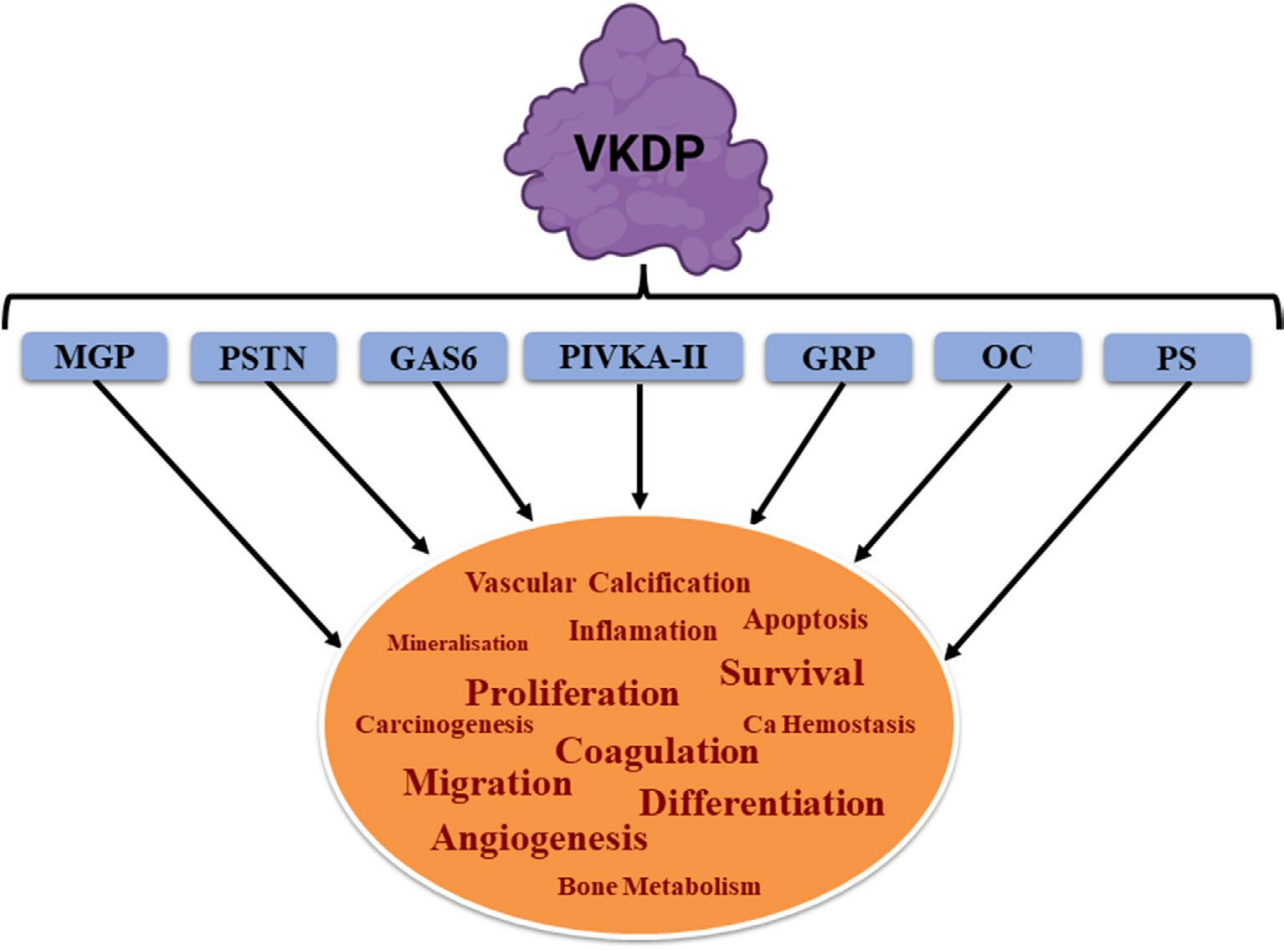
The diverse functions and implications of major cancer‐related VKDPs. These proteins, which require proper carboxylation, were initially studied for their hepatic roles in coagulation. However, expanding research revealed their involvement in various extrahepatic activities, including vascular calcification, bone metabolism, inflammation, carcinogenesis, apoptosis, calcium homeostasis, mineralization, bone growth control, cell migration, angiogenesis, and signal transduction.

Among VKDPs, growth arrest‐specific gene 6 (Gas6) [153], protein S (also called S‐Protein, ProS1, and PS) [154], periostin (PSTN) [155, 156], Gla‐rich protein (GRP) [157], matrix Gla protein (MGP) [158, 159], osteocalcin (OC) [160], and prothrombin induced by vitamin K absence or antagonist‐II (PIVKA‐II) [161] are the main cancer‐related VKDPs studied so far (Figure 5). Dahlberg et al. [83] extensively reviewed these VKDPs.

The following sections will include an outlook on the possible effects of vitamin K on cancer‐related processes, as well as detailed discussions on known cancer‐related VKDPs in both their carbonylated and uncarboxylated forms concerning cancer. It is believed that understanding these configurations could aid in further experiments exploring the potential CALU impacts on cancer status through the modulation of VKDP carboxylation.

#### 3.2.5 Vitamin K and Cancer‐Related Processes

The importance of vitamin K for the carboxylation of VKDPs and its effect on the various physiological processes, such as bone homeostasis, cardiac growth, and anti‐vascular calcification, has been evident [84, 162]. Despite some ambiguities in the effect of vitamin K on cancer, the dependence of cancer‐related activities on vitamin K, conducted by VKDPs, was shown in different studies. The dependence of GAS6 and PROS1 and PIVKA‐II cancer‐related activities on vitamin K was evident [163, 164]. A moderately suppressive effect of vitamin K2 on hepatocellular carcinoma recurrence [165, 166] and on cancer cell lines of cholangiocellular carcinoma, ovarian, and pancreatic cancers was also reported [167, 168, 169]. Moreover, inhibition of tumor cell proliferation and differentiation, and induction of apoptosis and autophagy in tumor cells by vitamin K was demonstrated [170]. Accordingly, the potential impact of vitamin K on cancer‐related processes seems inevitable. Further details on the major cancer‐associated VKDPs, with emphasis on their carboxylation, which is probably regulated by CALU, are provided in the following sections.

Warfarin is a vitamin K antagonist that interferes with the γ‐carboxylation of glutamic acid residues in VKDPs [171]. Since both warfarin [172] and CALU acts as γ‐carboxylation inhibitors [7, 37, 39, 40, 75], it is conceivable that CALU is a regulator of warfarin sensitivity [38, 39]. This assumption was later confirmed by demonstrating the association between CALU polymorphism and the required dose of warfarin in the African‐American population [173]. The CALU overexpression in warfarin‐resistant rats was documented in previous studies [38], where it was identified as the VKOR regulator [74] and implicated in human warfarin sensitivity [174].

Carboxylation of cancer‐related VKDPs was also shown to be inhibited by warfarin [175, 176]. In this regard, the use of warfarin for controlling the functions of VKDPs that contribute to cancer‐related processes has been proposed [152, 177] (See next section).

#### 3.2.6 The Main Cancer‐Related VKDPs

##### 3.2.6.1 GAS6

GAS6 is produced in various effector cells that contribute to TME [152]. Among cancer‐associated VKDPs, GAS6 acts as a ligand for three receptor tyrosine kinases (RTKs), including Tyro3, Axl, and Mertk (TAMs) [178]. TAMs are involved in inflammation, cancer progression, and autoimmunity [179, 180]. Together with TAMs, GAS6 can activate the kinase activity [181, 182] and regulate proliferation, migration, differentiation, adhesion, apoptosis, and survival of cancer cells [183].

In TME, it has been suggested that tumor cells train infiltrating leukocytes (mainly tumor‐associated macrophages) to produce high levels of GAS6, as a reinforcing loop, promoting tumor growth [184]. The survival effect of γ‐carboxylated GAS6 on the endothelium by protecting endothelial cells from apoptosis, utilizing classical intracellular signaling pathways, was previously reported [185]. The impact of GAS6 on the response to chemotherapy of cancer patients and its association with this response was also evident [177, 186, 187, 188]. Besides, the overexpression of Gas6 and the members of the TAM protein family in various cancers was shown, both in vitro and in vivo [189, 190, 191]. Accordingly, both TAM and GAS6 have been suggested as cancer therapeutic targets [192, 193]. In another investigation, by blocking the carboxylation of Gas6, warfarin could prevent the interaction of Gas6 with external phosphatidylserine on the surface of apoptotic cells and its residues in Gas6/TAM‐dependent tumors [176]. This approach could provide opportunities to target a wide range of Gas6/TAM‐dependent tumors. Inhibition of Gas6 function by warfarin was also investigated in melanoma, pancreatic, lung, and breast cancer models [194, 195, 196]. Furthermore, in preclinical studies, low‐dose warfarin treatment could prevent Axl receptor activation by Gas6 [197].

##### 3.2.6.2 PROS1

PROS1 is another VKDP plasma glycoprotein, which was primarily described as an anticoagulant cofactor, to facilitate the action of activated protein C on its substrates, including activated factor V (FVa) and activated factor VIII (FVIIIa) [198, 199]. Similar to Gas6, PROS1 also functions as a ligand for TAMs [178]. In a more recent study, PROS1 was introduced as a tumor‐derived functional ligand for Tyro3 that supports cancer cell survival [200]. The involvement of PROS1, as a ligand for the TAM receptors, was described in several vital biological processes, comprising phagocytosis of apoptotic cells, cell survival, activation of innate immunity, and vessel integrity [154, 163]. The physiological importance of Pros1 in the development and progression of different cancers was indicated [201, 202, 203, 204, 205, 206]. PROS1 and GAS6 share 44% amino acid sequence identity and have similar domain organizations [207, 208]. GAS6 and PROS1 are among the VKDPs whose functions are critical in cancer‐related processes. Due to the involvement of TAMs and their ligands GAS6 and PROS1 in oncogenic processes such as cell survival, invasion, migration, chemotherapy resistance, and metastasis, whereby their expression is often associated with poor clinical outcomes, new strategies have been developed to target the TAM ligands for cancer therapy [209]. In this regard, TAM inhibitors have been considered for anti‐tumor therapeutic responses [152].

##### 3.2.6.3 PSTN

Another cancer‐associated VKDP is PSTN, which is the most abundant Gla‐containing VKDP, expressed by most normal adult tissues [210], and reported to be secreted by mesenchymal stromal cells, where it was shown to be inhibited by warfarin [174]. PSTN is an adhesion molecule that acts during bone and tooth formation and maintenance [55, 211]. Physiologically, PSTN regulates embryonic formation, ECM structure, and other collagen‐rich connective tissues [212], and tendons [213]. PSTN is involved in many biological events such as cell migration [214, 215], angiogenesis, and myocardial remodeling [216], connective tissue development and maintenance [217], tissue repair and regeneration [218], collagen fibrillogenesis, cell survival [219], cell proliferation, tumor angiogenesis, and metastasis [220, 221].

The PSTN's abnormal upregulation has been observed in multiple pathological processes, such as inflammatory diseases and fibrosis [222, 223, 224], and has been associated with increased cell migration, chemoresistance, and poor prognosis in various human cancers [215, 225, 226, 227, 228, 229, 230, 231, 232, 233]. The involvement of PSTN in driving oncogenesis and metastasis in multiple tumors, including gastric tumors [234], BC [235, 236], papillary thyroid carcinoma, HNSC [237], and other cancers has also been reported [156]. PSTN has been suggested as a chemoattractant for cancer cells, a key factor in metastatic colonization, by conditioning the premetastatic niche [215, 238, 239]. PSTN has been shown to promote cellular migration and invasion by modulating EMT via the ERK signaling pathway [155, 240]. A mechanism for the production and induction of cytokines/chemokines from cancer cells by PSTN to promote the mobilization and differentiation of M2 macrophages and activate the CAFs via integrin‐dependent NF‐κB and TGF‐β2 signaling, resulting in enhanced cell growth and metastasis, was suggested [241].

##### 3.2.6.4 GRP

GRP, as a member of VKDPs with a relatively high Gla residue density (16 Gla residues), participates in stabilizing the cartilage matrix, chondrogenesis, and inhibition of osteogenesis [242, 243, 244]. In both the GRP knockdown and warfarin‐exposed zebrafish, irreversible growth retardation and altered skeletal development are evident [245]. GRP is a circulating protein associated with pathologic calcification in the skin and arteries, and accumulates at sites of aberrant calcification [246]. This protein has been suggested as a negative regulator of osteogenic differentiation [247]. GRP acts as a modulator of calcium availability in the ECM [243, 246], and an inhibitor of calcification in the cardiovascular and articular systems [114]. It has a similar inhibitory effect on calcification and inflammation processes [245]. Increased levels of uncarboxylated GRP (ucGRP) are associated with pathological calcification‐related diseases [114], osteoarthritis [248, 249], and tumors in the breast and skin [157].

##### 3.2.6.5 MGP

MGP, another secretory VKDP with 84 amino acid residues, is found in a variety of tissues, including bone matrix [250], vascular smooth muscle cells [251], arterial wall [252], heart, lungs, and kidneys [253], venous wall [254], dental cementum [255], trabecular meshwork cells [256], and a wide variety of tissues during the embryonic period [253, 254, 255]. Under normal physiological conditions, the carboxylated MGP (cMGP) inhibits calcification [257]. The MGP upregulation in BC cells [240], primary renal carcinoma [258], testicular and prostatic carcinomas [259], ovarian cancer [260], and GC [261], glioma [251, 262, 263], glioblastoma [251, 264], and its downregulation in colorectal adenocarcinomas and malignant colorectal cells [265] and during the progression of lung cancer [266], and in renal and prostate carcinoma [258] was shown.

MGP is associated with cell differentiation and proliferation, migration, invasion, survival, tumoral development, and metastasis [257, 258, 259, 263]. The intracellular MGP was reported to promote GC cell proliferation and survival by acting as a transcriptional co‐activator of STAT5 [162]. Therefore, it has been suggested as a suitable marker for tumor development, prognosis, diagnosis, and treatment in various cancers, although the effect of MGP may be tumor‐type‐dependent.

##### 3.2.6.6 OC

OC, or Bone Gla‐Protein (BGP), with 49 amino acids [267], which constitutes 20% of the noncollagenous protein of bones, is a product of osteoblasts [268] and odontoblasts, the first identified extrahepatic Gla protein [249, 269]. Carboxylated OC (cOC) is a key factor in bone metabolism [270], and together with uncarboxylated OC (GluOC) plays a role in glucose metabolism [271]. GluOC is also involved in neural development and the male reproductive tract, and has a relationship with tumorigenesis [272]. GluOC has been shown to have a tumor‐promoting effect on pancreatic cancer [273]. The correlation between the level of circulating OC‐positive cells and bone metastasis in patients with breast cancer has been evident in independent studies [274, 275, 276]. Xu et al. [277] showed that GluOC facilitates the proliferation and metastasis of MDA‐MB‐231 cells by accelerating the transforming growth factor‐β (TGF‐β)/SMAD3 signaling pathway. They also showed that GluOC stimulates the expression of the IL‐8 and PTHrP genes in the same cells, both of which can act as osteolytic factors, and suggested GluOC as a novel therapeutic target to prevent or treat TNBC bone metastasis in the clinic. As such, the effect of GluOC in animals is still unclear, and further experimental verification in vivo is needed.

##### 3.2.6.7 PIVKA‐II

PIVKA‐II (des‐γ‐carboxy prothrombin) is an abnormal prothrombin molecule, generated as a result of an acquired defect in the posttranslational γ‐carboxylation of the prothrombin precursor in malignant cells, in the absence of vitamin K or when a vitamin K antagonist is used [278]. PIVKA‐II is significantly increased in the serum of hepatocellular carcinoma (HCC) patients, and it could serve as a new serum marker in this regard [279, 280]. The overproduction of prothrombin precursor in tumor cells, deficiencies in the vitamin K dependent carboxylation, and vitamin K deficiency in tumor tissue have been suggested to be the underlying mechanisms of PIVKA‐II production in HCC [164].

#### 3.2.7 The Impact of CALU on Cancer Treatments

##### 3.2.7.1 The CALU Correlations With the Cancer Cell Responsiveness to Chemotherapy

One of the main problems of cancer patients is the inherent or acquired resistance to therapies, which generally leads to cancer cells gaining the ability to escape the effects of chemotherapy, increasing relapse rates, reducing overall survival, and finally, patient death [281]. Drug resistance is a complex and multifactorial event, for which several potential mechanisms that involve the activation or suppression of multiple biochemical pathways have been identified [282]. The relationship between CALU expression and drug resistance in cancer patients has been demonstrated [56]. Lin and colleagues [50], who studied MES‐SA and MES‐SA/Dx5 (doxorubicin‐resistant) uterine cancer cells, found CALU to be an upregulated protein in MES‐SA/Dx5 cells, while it was downregulated in MES‐SA cells treated with doxorubicin. In separate work on A431 cancer cells and the cisplatin‐resistant strain (A431/Pt), upregulation of CALU in the resistant strain was shown [283]. Some types of leukemia and various human tumors, in particular solid tumors, are sensitive to As_2_O_3_, and the downregulation of CALU was observed where the sensitivity to As_2_O_3_ was increased in the presence of Emodin [284].

Following the sensitivity analysis of common chemotherapeutic medications against cells, a correlation between CALU expression, tumor microenvironment, and the sensitivity of the ccRCC patients to common chemotherapeutic and immunotherapy drugs was also shown. Accordingly, it was demonstrated that elevated CALU expression causes drug resistance to chemotherapy drugs such as Embelin, Salubrinal, and Tipifarnib, and increased sensitivity to chemotherapeutic drugs such as 5Z‐7‐Oxozeaenol, AMG‐706, and Cytarabine [99].

There is currently no clear explanation for how CALU affects medication resistance. Nonetheless, the impact of CALU on treatment resistance can be explained in light of the available data on its impact on calcium homeostasis. Because of the disruption of calcium homeostasis after ERS, cells evade apoptosis, which is the cause of drug resistance [62, 285]. Given CALU's critical role in maintaining intracellular Ca homeostasis, variations in its expression may have an impact on apoptosis or cell survival by altering calcium homeostasis. Further investigations are needed to determine whether the levels of particular CALU isoforms or the total expression level of CALU are significant in this situation.

As previously mentioned, CALU is a carboxylation inhibitor that directly affects multiple VKDPs, which in turn can influence drug resistance. This includes the impact of GSA6 on the response to cancer chemotherapy [177, 186, 187, 188] and the association of PSTN overexpression with enhanced cell migration, chemoresistance, and poor prognosis in various human cancers [215, 225, 226, 227, 228, 229, 230, 231, 232, 233]. Therefore, it is conceivable that CALU might also affect drug resistance indirectly by regulating the carboxylation of effector VKDPs.

##### 3.2.7.2 The CALU Correlation With the Immune‐Related Pathways and Cancer Cell Responsiveness to Immunotherapy

There is evidence for the association of CALU with immune‐related pathways in various cancer treatments. Immune landscape characterization in clear cell renal cell carcinoma (ccRCC) revealed that CALU expression is positively associated with neutrophils and macrophages [99]. In contrast, it is negatively associated with natural killer T cells and CD8+ T cells, although these cells, besides monocytes/macrophages, were found as binding targets of CALU.

In our previous work, we showed that CALU expression in CRC is positively correlated with the infiltration of multiple immune cell types, including B cells, CD8+ T cells, CD4+ T cells, macrophages, neutrophils, and dendritic cells [100]. A positive correlation between CALU expression and CAF infiltration was observed, along with its involvement in the EMT process in both CAFs and malignant cells in various cancer types [128].

Beyond its association with CAFs, Du et al. [48] also demonstrated CALU's correlation with other tumor‐infiltrating immune cells (e.g., CD8+ T cells and macrophages), immune receptor activity, cytokine binding, T cell receptor signaling, and multiple ICRGs, including PD‐1, PD‐L1, PD‐L2, CTLA‐4, and TIGIT, as well as patient response to immunotherapy. They showed that high CALU expression significantly increased responsiveness to immune checkpoint blockade (ICB) therapy. The correlation of CALU with the immune system was also demonstrated by the identification of CALU as a potential negative regulator of the virus‐induced type I interferon activation [286]. Therefore, it can be inferred that CALU expression is associated with immune cell infiltration, indicating potential interactions between CALU and the immune system in the tumor microenvironment. Many other studies have also supported these findings and suggested a role for CALU in immune‐based therapeutic strategies in cancer [48, 107, 127].

## 4 Conclusion

In this review, we highlighted the imperative features of CALU, including its genetics, structure, and expression characteristics in different conditions and tissues, as well as its diverse and extensive functions. CALU has been shown to mediate multiple Ca^2+^‐dependent functions, including the regulation of ER/SR membrane proteins (VKOR, γC, RYR, and SERCA2a), involvement in ERS, and facilitation of proper structural modeling, maturation, folding, and sorting of membrane and secretory proteins. These activities facilitate the contribution of CALU to cellular processes, such as cell cycle modulation, proliferation, cell morphology, signal transduction, contraction, exocytosis, apoptosis, and ferroptosis, and the production and maintenance of the ECM. Besides, due to its Ca^2+^‐binding tendency, CALU is associated with normal calcification processes, such as cartilage cell development, bone metabolism, and fracture resorption. In this regard, it is also associated with cystic fibrosis, cardiovascular and neuromuscular disorders, and other pathological Ca deposits, such as lung fibrosis, vascular thrombosis, and atherosclerosis.

The implications of CALU in cancer‐related processes, such as cancer development, TME, and promoting malignant phenotypes, including enhanced cell survival, invasiveness, filopodia formation, cell migration, and metastasis, have also been shown. Besides, associations between CALU expression and a wide range of malignancies were evident. Accordingly, CALU has been introduced as a hallmark of EMT. CALU can also influence cancer‐related processes through its regulatory role in the γ‐carboxylation of VKDPs associated with cancer, such as PSTN, GRP, Gas6, PS, and MGP. The correlations of these proteins with multiple pathological processes, including calcifications, autoimmunity, and inflammatory diseases, highlight the indirect involvement of CALU in various pathological conditions.

CALU expression in cancer cells not only governs intracellular processes but also potentially influences the immune response within the tumor microenvironment. The relationship of CALU with cancer patients' responsiveness to various therapy methods, including chemotherapy and immunotherapy, has also been proven. In this regard, the CALU association with immune response, its correlation with CAFs and other tumor‐infiltrating immune cells, and its involvement in various immune‐related pathways, such as immune receptor activities, cytokine binding, the T cell receptor pathway, multiple ICRGs, and patients' responsiveness to immunotherapy, have been demonstrated.

Considering the involvement of CALU in diverse physiological processes, its key role in cellular Ca homeostasis, and especially its inhibitory role in several important Ca^2+^‐dependent activities, and the opposing and pleiotropic functions of its isoforms, which are sure to play a balancing role in creating cellular homeostasis, it is considered a crossroads between normal cellular processes and the operational pathways of normal cellular systems such as coagulation, calcification, etc., as well as many inflammatory diseases and cancer.

Concerning the reported association of CALU with cancer therapy responsiveness and different immune pathways, it is assumed that the CALU isoforms must play essential roles in these pathways. Concerning the proposed modulating function of CALU on cancer‐related processes, further investigations are required to elucidate the impacts of each of the individual CALU isoforms on the γ‐carboxylation process, immune pathways, and cancer‐related processes.

The contradicting actions of CALU isoforms in cancer‐related processes demonstrate a dual behavior of this protein, indicating the modulatory roles of the isoforms in cellular processes, producing a state of equilibrium in the cell. The protein's extensive engagement in many cellular processes necessitates a balance in the ratio of CALU isoforms to achieve this cellular balance. Thus, it would appear that a regulatory mechanism is necessary to create such a balance among the transcript alternative splicing products. It has been established that the TRA2A regulates the alternative splicing of CALU in this respect. Gaining additional knowledge about these regulatory processes would help researchers better understand CALU‐related research and increase the likelihood that the desired CALU isoform or isoforms may be produced for use in cancer treatment.

## Author Contributions

The first draft of the manuscript was written by A. Zomorodipour and Parinaz Nasri, and all authors commented on previous versions of the manuscript. All authors read and approved the final manuscript.

## Ethics Statement

The authors have nothing to report.

## Consent

The authors have nothing to report.

## Conflicts of Interest

The authors declare no conflicts of interest.

## Data Availability

The authors have nothing to report.
